# Outcome and patterns of failure after postoperative intensity modulated radiotherapy for locally advanced or high-risk oral cavity squamous cell carcinoma

**DOI:** 10.1186/1748-717X-7-175

**Published:** 2012-10-22

**Authors:** Andreas Geretschläger, Beat Bojaxhiu, Susanne Crowe, Andreas Arnold, Peter Manser, Wock Hallermann, Daniel M Aebersold, Pirus Ghadjar

**Affiliations:** 1 Department of Radiation Oncology and Division of Medical Radiation Physics, Bern University Hospital, Freiburgstrasse, 3010, Bern, Switzerland; 2 SAKK Coordinating Center, Effingerstrasse 40, 3008, Bern, Switzerland; 3 Department of Otorhinolaryngology, University Hospital Bern, Freiburgstrasse, 3010, Bern, Switzerland; 4 Department of Craniomaxillofacial Surgery, University Hospital Bern, Freiburgstrasse, 3010, Bern, Switzerland

**Keywords:** Head and neck cancer, IMRT, Oral cavity, Outcome, Postoperative

## Abstract

**Background:**

To determine the outcome and patterns of failure in oral cavity cancer (OCC) patients after postoperative intensity modulated radiotherapy (IMRT) with concomitant systemic therapy.

**Methods:**

All patients with locally advanced (AJCC stage III/IV) or high-risk OCC (AJCC stage II) who underwent postoperative IMRT at our institution between December 2006 and July 2010 were retrospectively analyzed. The primary endpoint was locoregional recurrence-free survival (LRRFS). Secondary endpoints included distant metastasis-free survival (DMFS), overall survival (OS), acute and late toxicities.

**Results:**

Overall 53 patients were analyzed. Twenty-three patients (43%) underwent concomitant chemotherapy with cisplatin, two patients with carboplatin (4%) and four patients were treated with the monoclonal antibody cetuximab (8%).

At a median follow-up of 2.3 (range, 1.1–4.6) years the 3-year LRRFS, DMFS and OS estimates were 79%, 90%, and 73% respectively. Twelve patients experienced a locoregional recurrence. Eight patients, 5 of which had both a flap reconstruction and extracapsular extension (ECE), showed an unusual multifocal pattern of recurrence. Ten locoregional recurrences occurred marginally or outside of the high-risk target volumes. Acute toxicity grades of 2 (27%) and 3 (66%) and late toxicity grades of 2 (34%) and 3 (11%) were observed.

**Conclusion:**

LRRFS after postoperative IMRT is satisfying and toxicity is acceptable. The majority of locoregional recurrences occurred marginally or outside of the high-risk target volumes**.** Improvement of high-risk target volume definition especially in patients with flap reconstruction and ECE might transfer into better locoregional control.

## Background

Treatment of locally advanced or high-risk oral cavity cancer (OCC) involves extensive surgical procedures, often combined with flap reconstruction, followed by postoperative radiotherapy (RT) with systemic therapy if certain risk factors are present. Postoperative RT as compared to preoperative RT has been shown to increase locoregional control of patients with locally advanced head and neck cancer [1]. In addition postoperative RT increased overall survival (OS) in nodal positive patients as compared to surgery alone [2]. Furthermore postoperative cisplatin based chemoradiation is known to increase time to locoregional recurrence as well as OS in patients with positive resection margins and lymph node metastasis with extracapsular extension (ECE) [3]. Recently, the development of intensity modulated RT (IMRT) significantly reduced xerostomia when compared to 3-dimensional conformal RT in a randomized controlled trial [4,5] and swallowing sparing IMRT has been described to potentially reduce RT related dysphagia [6].

In 2006/2007 we reevaluated our institutional policy for IMRT treatment of OCC patients. Having started IMRT treatments in 2002, we could draw upon own experience and patient results as well as a small number of publications [7-13]. We then adapted our institutional guidelines for treatment and follow-up with a special focus on target volume definition and dose prescription. We have now retrospectively analyzed the outcome and patterns of failure of patients at our center having been treated accordingly.

## Methods

### Patient selection

We retrospectively identified all patients with newly diagnosed OCC with either locally advanced disease (American Joint Committee on Cancer (AJCC) stage III or IV) or early stage high-risk disease (positive or close margins (< 3mm); perineural invasion; lymphovascular space invasion and synchronous primary tumors) who underwent postoperative IMRT between December 2006 and July 2010 at our Department of Radiation Oncology, Inselspital, Bern University Hospital. Patients with a history of another malignancy within 5 years, histology other than squamous cell carcinoma, distant metastatic disease or prior neoadjuvant therapy were excluded. Patients were staged according to the AJCC 2002 guidelines [14]. This study was approved by the local ethics committee.

### Treatment

Prior to start of any treatment, stage of disease, sequence of treatment and modalities were defined in the weekly interdisciplinary institutional head-and-neck tumorboard for all patients.

#### Surgery

All patients underwent resection of the primary tumor and an uni- or bilateral modified radical neck dissection in the Department of Otorhinolaryngology, Head and Neck Surgery and / or Department of Craniomaxillofacial Surgery, Inselspital Bern, University Hospital. In cN+ patients aim of surgery was complete resection of the primary tumor and of all cervical metastastic lymph nodes. The decision to do selective neck dissections in cases with cN0 or in the contralateral node negative neck was at the discretion of the head and neck surgeon.

#### Radiotherapy

Prior to IMRT planning all patients were referred for dental evaluation and treatment if necessary. For treatment planning a dedicated high-resolution CT scan with 3 mm slices and intravenous contrast was used. Patients were immobilized in the supine position using a thermoplastic mask covering head and shoulders. A bite block / tongue depressor was used to separate upper from lower jaw, all patients were additionally told to extend their (remaining) tongue to reach the backside of their lower jaw incisors and keep the tongue in this position during treatment if possible. All visible surgical scars were marked with flexible wires.

It was our policy to contour the resected tumor and all metastatic lymph nodes in their former location and full dimension in the planning CT if possible, using all available information (clinical descriptions, all preoperative imaging modalities, surgical reports). Additional image fusion of preoperative MRI or PET-CT was performed if deemed useful. Three different risk levels of clinical target volume (CTV) were defined. CTV72 was defined as gross tumor either due to early postoperative tumor progression with an additional isotropic margin of 1.5 cm and treated to 72 Gy. CTV60-66 was defined as the former tumor bed (FTB), areas with microscopically incomplete resection (R1) and all neck levels having harboured lymph node metastasis with ECE with an additional isotropic margin of 1.5 cm. In case of reported intraoperative spillage (tumor and / or metastatic lymph node rupture) the whole corresponding surgically manipulated area was defined as CTV60-66, e.g. the complete area of neck dissection [15]. All of CTV60-66 was treated to 66 Gy except in a few cases of R0 resection of the primary tumor with generous margins (> 5 mm), where the FTB was treated to 60 instead of 66 Gy. CTV54 included the CTV72 or CTV60-66 plus dissected lymph node levels with 1 (pN1) or more metastatic lymph nodes (pN2b, pN2c) without ECE and the elective contralateral not dissected neck levels in case of unilateral neck dissection and was treated to 54 Gy. The definition of elective nodal target volumes followed the recommendations proposed by Eisbruch et al. [16]. All prescribed CTV-margins were anatomically adapted to account for natural barriers such as thyroid and cricoid cartilage, hyoid, mandibular or vertebral bone and for skin and air. The resulting CTVs were finally expanded to planning target volumes (PTVs) by adding a symmetric 3 mm margin for setup error compensation. In a final adjustment all PTVs were set back from the skin surface 3–5 mm to allow for dose build-up except where the skin was deemed to be at risk of harbouring microscopic tumor e.g. the neck dissection scar in case of tumor spillage. Instead of using a skin flap in these cases we tried to optimize dose build-up by putting more weight on tangential beam directions accepting minor under-dosages. All planning PTVs were treated sequentially in a “shrinking-volume” technique with a fractionation of 5 times 2 Gy per week, resulting in two or three treatment plans per patient. Dose prescription was done to the median dose D50% of the PTV in accordance with the ICRU report 83. All treatment plans were contoured and calculated by Eclipse treatment planning system (Varian Medical Systems, Palo Alto, CA).

#### Concomitant systemic therapy

The standard concomitant therapy consisted of cisplatin 100mg/m2 day1 in three week intervals for all patients. Patients not deemed fit for cisplatin chemotherapy because of pre-existing co-morbidities or poor overall performance status were occasionally evaluated for treatment with either carboplatin or the monoclonal antibody cetuximab in an individual therapeutic approach by the responsible medical oncologist knowing that there is until present no sufficient data to prove the benefit of this treatment.

### Assessments and evaluations

After RT all patients underwent follow-up visits on a regular basis. These visits were scheduled every 3 months for the first 2 years, twice a year until the 5th year and yearly thereafter. For this study the follow-up information closeout date was September 2011. Eight to twelve weeks after the end of treatment a post-therapy baseline CT or MRI was performed.

Time-to-event endpoints were calculated from end of RT until the date of event. Patients not experiencing an event were censored at the date of the last follow-up visit.

Toxicities were graded according to the National Cancer Institute (NCI) Common Terminology Criteria for adverse events (CTCAE) version 3.0. The symptoms pain, dermatitis, mucositis, dysphagia, xerostomia and osteonecrosis were assessed. Acute toxicity was defined as complications during RT and until 3 months after end of RT; late toxicity as complications occurring thereafter.

### Analysis of recurrences

The recurrent or persistent tumor volume as defined by all available diagnostic images, was used to categorize the recurrences as “in-volume”, if >95% of the recurrent locoregional tumor volume was within the corresponding CTV54, CTV60-66 or CTV72; “marginal”, if 20–95% was within the CTV54, CTV60-66 or CTV72; or “out-volume”, if <20% of the recurrent tumor volume was within the respective CTV, as previously described [17,18]. The relation of all recurrences to both CTVs was assessed sequentially.

### Statistical considerations

The primary endpoint was locoregional recurrence-free survival (LRRFS). Secondary endpoints included distant metastasis-free survival (DMFS), OS, acute and late toxicity. Late toxicity at last follow-up visit was also assessed to determine whether the late toxicity persisted or was transient.

Time-to-event endpoints were estimated using the Kaplan Meier method. Univariate and multivariate analyses were performed using Cox proportional hazards models and the backward selection method (criterion for removal: *p* ≥ 0.05). To be assessed in the multivariate analysis, a variable had to be significant (*p* ≤ 0.1) in the univariate analysis. Categorical variables were summarized using absolute and relative frequencies; continuous variables by descriptive statistics. P-values were two-sided, not adjusted for multiple testing, and considered significant if < 0.05. The data were analyzed in SPSS (SPSS Inc., Chicago, IL, version 19.0) and SAS (Statistical Analysis Systems Institute Inc, version 9.2).

## Results

### Patient and treatment characteristics

In total 53 patients were retrospectively analyzed. Seven and thirty patients had AJCC stage III (13%) or IV (57%), in the remaining 16 patients with AJCC stage I/II (30%), the following risk factors were present: positive margins (*n*=2), close margins (*n*=9), perineural invasion (*n*=3), lymphovascular space invasion (*n*=2) and 1 patient had 2 synchronous primary tumors (buccal mucosa and tongue). Pre-treatment staging of the head and neck was performed by either CT (*n*=33), MRI (*n*=12) or both (*n*=8). Prior to treatment, a PET-CT scan was used on 9 patients (17%). A total of 17 patients were ECE positive. Twenty-nine patients (55%) underwent soft tissue reconstruction using either a local musculocutaneous pedicled flap (*n*=17), regional musculocutaneous pedicled flap (*n*=6) or free flap (*n*=6). ECE was detected in 11 flap reconstruction cases. Further patient and treatment characteristics are summarized in Table 1. Median time between surgery and RT was 6.7 (range, 3–17) weeks.

**Table 1:** Patient and treatment characteristics (n = 53)

**Characteristics**	**n (%)**
Age (years)#	
≤ 60	25 (47)
> 60	28 (53)
Gender	
Female	17 (32)
Male	36 (68)
Karnowsky PS	
> 70	42 (79)
≤ 70	11 (21)
Site	
Tongue	22 (41)
Floor of mouth	17 (32)
Alveolus and Gingiva	11 (21)
Others*	3 (6)
Tumor classification	
pT1	8 (15)
pT2	25 (47)
pT3	5 (10)
pT4	15 (28)
Nodal classification	
cN0**	2 (4)
pN0	18 (34)
pN1	10 (19)
pN2	22 (41)
pN3	1 (2)
Grading	
Moderate (G2)	37 (70)
Poor (G3)	16 (30)
Resection margins	
R0	15 (28)
Close (< 3mm)	25 (47)
R1	13 (25)
Surgery to primary tumor	
Wide local excision	9 (17)
Partial glossectomy	18 (34)
Hemiglossectomy	4 (7)
Mandibular resection	4 (7)
Partial maxillectomy	3 (6)
Composite oral cavity resection	15 (29)
Neck dissection##	
None	2 (4)
Ipsilateral	28 (53)
Bilateral	23 (43)
Gastrostomy tube	
None	28 (53)
Used	25 (47)

Abbreviations: *PS* performance status; # median 60 years (range, 40–88 years); * buccal mucosa n=2 and hard palate n=1; **Two patients (4%) did not undergo a neck dissection; ## Of the 51 patients who underwent a neck dissection a median of 44 nodes (range, 14–101 nodes) were removed.

Median total RT dose was 66 (range, 60–72) Gy. Two patients received 72 Gy as there was evidence of macroscopic neck metastasis during treatment planning.

Twenty-five patients (47%) underwent concomitant chemotherapy (cisplatin n=23, carboplatin n=2). The median number of chemotherapy cycles was 3 (range, 2–3). Four patients (8%) underwent treatment with the monoclonal antibody cetuximab. The median follow-up was 2.3 (range, 1.1–4.6) years for the surviving patients.

### Locoregional recurrence

**Figure 1:**
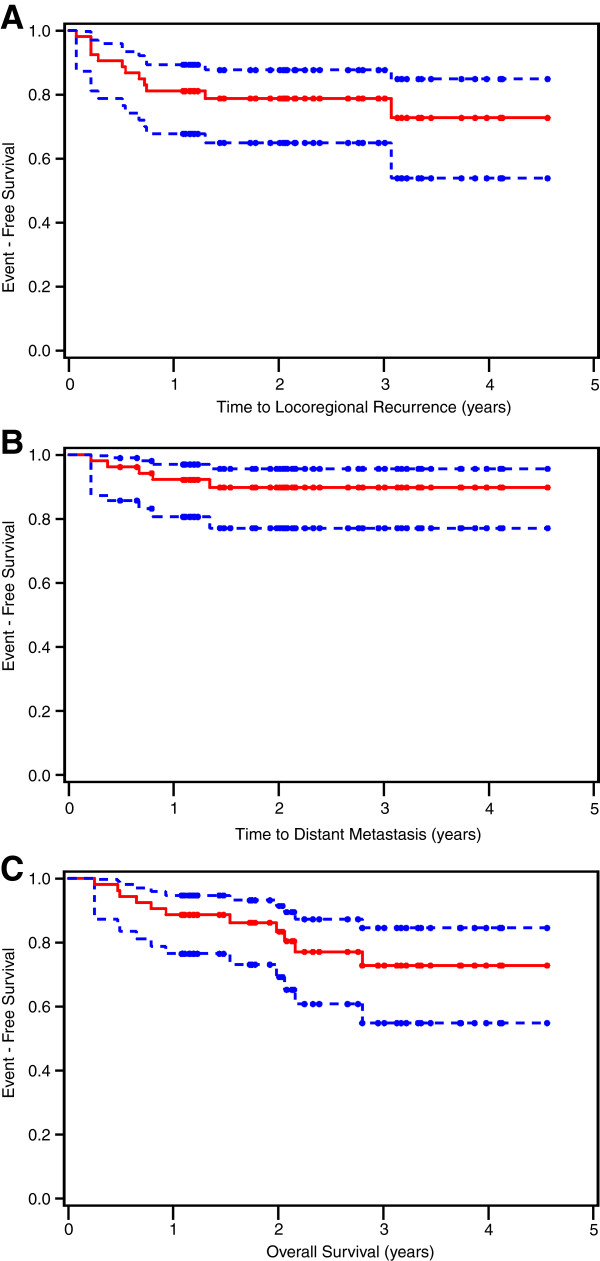
Locoregional recurrence-free survival (LRRFS) (A), distant metastasis-free survival (DMFS) (B) and overall survival (OS) (C).

At the time of analysis, 9 local recurrences and 9 regional recurrences were observed in 12 patients. Of those patients, 3 had only local recurrence, three had only regional recurrence and 6 had both. The median time to locoregional recurrence was not reached. The 2- and 3-year LRRFS estimates were both 79% (Figure 1A). In a multivariate Cox proportional hazards model, N-classification > N1 remained a significant prognostic factor for LRRFS (hazard ratio (HR): 4.7; 95% Confidence interval (CI): 1.3–17.4; *p*=0.02)) (Table 2).

**Table 2:** Treatment outcome analysis

**Factor**	**Associated level**	**Cox regression analysis hazard ratio (95% CI) (p-value)**		
		**LRRFS**	**DMFS**	**OS**
*Univariate analysis*				
Age (years)	>60	1.31 (0.42, 4.13) (0.64)	1.44 (0.24, 8.62) (0.69)	2.46 (0.65, 9.28) (0.18)
Sex	Male	0.92 (0.27, 3.07) (0.89)	0.75 (0.12, 4.48) (0.75)	1.81 (0.39, 8.44) (0.45)
KPS	≤70	1.20 (0.32, 4.44) (0.79)	0.94 (0.11, 8.46) (0.96)	0.74 (0.16, 3.42) (0.70)
T-classification	pT_3-4_	1.73 (0.56, 5.37) (0.34)	2.44 (0.41, 14.58) (0.33)	1.43 (0.44, 4.70) (0.55)
N-classification	pN_2-3_	4.71 (1.27, 17.45) (0.02)	114.6 (0.06, >1000) (0.22)	4.65 (1.23, 17.63) (0.02)
AJCC stage	IV	4.43 (0.97, 20.27) (0.05)	56.7 (0.04, >1000) (0.27)	4.13 (0.90, 19.10) (0.07)
Grading	G3	1.82 (0.58, 5.73) (0.31)	3.4 (0.57, 20.4) (0.18)	0.89 (0.24, 3.40) (0.87)
Resection margins	R1	0.65 (0.14, 2.98) (0.58)	0.78 (0.09, 6.96) (0.82)	1.73 (0.50, 5.93) (0.38)
ECE positive LN	yes	2.29 (0.74, 7.14) (0.15)	3.54 (0.58, 21.4) (0.17)	2.19 (0.66, 7.27) (0.20)
Chemotherapy*	yes	0.56 (0.18, 1.75) (0.32)	1.15 (0.19, 6.89) (0.88)	0.44 (0.13, 1.50) (0.20)
Flap reconstruction	yes	1.92 (0.58, 6.42) (0.29)	59.4 (0.04, >1000) (0.27)	2.48 (0.66, 9.35) (0.16)
Bilateral ND	yes	0.38 (0.10, 1.40) (0.15)	0.74 (0.12, 4.46) (0.75)	0.59 (0.17, 2.01) (0.40)
Gastrostomy tube	yes	1.68 (0.53, 5.30) (0.38)	1.70 (0.28, 10.17) (0.56)	1.03 (0.31, 3.38) (0.96)
Time OP to RT	> 6 weeks	0.59 (0.19, 1.89) (0.38)	0.73 (0.12, 4.37) (0.73)	0.68 (0.19, 2.35) (0.54)
*Multivariate analysis*				
N-classification	pN_2-3_	4.71 (1.27, 17.45) (0.02)	n.a.	4.65 (1.23, 17.63) (0.02)
AJCC stage	IV	1.68 (0.15, 18.51) (0.67)	n.a.	1.53 (0.14, 16.94) (0.73)

Abbreviations: *LRRFS* Locoregional recurrence-free survival, *DMFS* Distant metastasis-free survival, *OS* Overall survival, *OP* surgery, *KPS* Karnowsky performance score, *ECE* extracapsular extention, *LN* lymph node, *n.a.* not applicable, *ND* neck dissection, *including treatment with cetuximab.

The patterns of failure analysis revealed that 8 out of 12 locoregional recurrences showed an unusual multifocal pattern of recurrence, five of these patients had a flap reconstruction and ECE. Six of the total 12 locoregional recurrences occurred within the CTV54; 10 of the 12 marginally or outside of the CTV60-66 or CTV72 (Table 3).

**Table 3:** Localization and pattern of tumor recurrences in relation to the clinical target volumes

**Site of Primary**	**T**	**N**	**R**	**ECE**	**Flap**	**Systemic therapy**	**Time to recurrence (yrs)**	**Compartment of recurrence**	**Localization and pattern of recurrence**	**Relation of recurrence to CTV 54**	**Relation of recurrence to CTV60-66**
Floor of mouth	4	2b	c	Yes	Yes	Yes	0.2	Local	Outside FTB, not involving flap, multifocal	Marginal	Marginal
							0.8	Distant	Lung	n.a	n.a
Floor of mouth	1	2b	c	No	No	Yes	3.1	Local	Outside FTB, ipsilateral dorsal edge of tongue, unifocal	Marginal	Marginal
Floor of mouth	4	2b	c	Yes	Yes	Yes	0.7	Local	Within FTB and flap, multifocal	In	Marginal
							0.7	Distant	Lung	n.a	n.a
Lower alveolar ridge	4	2b	1	Yes	Yes	No	0.3	Local	Within FTB and flap, multifocal		
							0.3	Regional	Ipsilateral neck, multifocal	In	In
							0.4	Distant	Lung, Pleura, Skin, Liver	n.a	n.a
Tongue	2	2b	c	No	No	No	0.5	Local	Within FTB, unifocal	In	Marginal
							0.5	Regional	Bilateral neck (ipsilateral bulky), multifocal		
Tongue	2	2b	0	No	Yes	No	0.2	Local	Within FTB and flap, unifocal		
							0.2	Regional	Bilateral neck, multifocal	In	Marginal
							0.2	Distant	Pleura	n.a	n.a
Buccal mucosa	2	2b	1	Yes	Yes	Yes	0.1	Local	Within bed of surgical resection and flap, multifocal	Marginal	Marginal
							0.1	Regional	Contralateral neck, unifocal		
Tongue	3	2b	0	Yes	Yes	No	0.7	Local	Within FTB and flap, multifocal	In	Marginal
							0.7	Regional	Contralateral neck, unifocal		
Tongue	2	0	0	No	No	No	0.7	Local	Within FTB, unifocal	Marginal	Marginal
							0.7	Regional	Contralateral neck, multifocal		
Tongue	3	2c	c	Yes	Yes	Yes	0.7	Regional	Contralateral neck, unifocal	In	In*
Tongue	1	0	c	No	No	No	1.3	Regional	Contralateral neck, unifocal	Out	Out
Lower alveolar ridge, edge of tongue	4	0	0	No	Yes	No	0.5	Regional	Outside FTB, not involving flap, extending to ipsilateral base of skull and Fossa infratemporalis, unifocal	Out	Out

Abbreviations: *T* tumor classification, *N* nodal classification, *R* resection margin, *c* close (< 3 mm), *CTV* clinical target volume, *n.a.* not applicable;

*FTB* former tumor bed, *CTV* clinical target volume, *this patient was one of the two patients who received a total dose of 72 Gy, the regional

recurrence was inside the CTV72Gy.

### Distant metastasis

At the time of analysis 5 patients had developed distant metastasis, located in the lung, pleura, skin (*n*=1), bone and soft tissue (*n*=1), pleura (*n*=1) or lung only (*n*=2). All patients who developed metastasis had also either local or regional recurrence. The 2- and 3-year DMFS estimates were both 90% (Figure 1B). No clinical or pathological variable was found to be associated with distant metastasis (Table 2).

### Overall survival

Eleven patients (21%) died during follow-up; 9 due to cancer progression (cancer specific deaths), one patient due to cachexia and the other due to cardiac failure. The 2- and 3-year OS estimates were 83% and 73% (Figure 1C), and the respective cancer-specific survival rates were 85% and 82%. In a multivariate Cox proportional hazards model using backward selection, N-classification > N1 was the only significant prognostic factor for OS (HR: 4.6, 95% CI: 1.2–17.6; *p*=0.02) (Table 2).

### Pre-RT morbidity and acute and late toxicity

After surgery and prior to RT, pre-RT morbidity was determined (Table 4). Six patients (11%) were identified with pre-RT grade 2 pain, 17 (32%) had pre-RT grade 2 dysphagia and 7 (14%) had pre-RT grade 3 dysphagia.

**Table 4:** Pre-treatment morbidity and acute and late toxicity

		**Pre-Tx**	**Acute†**	**Late‡**	**Last late§**
Toxicity	Grade	n (%)	n (%)	n (%)	n (%)
Pain	0	31 (59)	1 (2)	42 (80)	47 (89)
	1	16 (30)	12 (23)	4 (7)	0
	2	6 (11)	32 (60)	5 (9)	5 (9)
	3	-	8 (15)	2 (4)	1 (2)
Dermatitis	0	53 (100)	-	51 (96)	53 (100)
	1	-	14 (26)	1 (2)	-
	2	-	30 (57)	1 (2)	-
	3	-	9 (17)	-	-
Mucositis	0	53 (100)	-	50 (94)	53 (100)
	1	-	7 (13)	1 (2)	-
	2	-	27 (51)	1 (2)	-
	3	-	19 (36)	1 (2)	-
Dysphagia	0	25 (47)	5 (9)	28 (53)	35 (66)
	1	4 (7)	5 (9)	4 (7)	7 (14)
	2	17 (32)	25 (48)	16 (31)	6 (11)
	3	7 (14)	18 (34)	5 (9)	5 (9)
Xerostomia	0	53 (100)	37 (70)	34 (64)	40 (75)
	1	-	13 (24)	15 (29)	10 (19)
	2	-	3 (6)	4 (7)	3 (6)
	3	-	-	-	-
Osteonecrosis	0	53 (100)	52 (98)	51 (96)	53 (100)
	1	-	-	-	-
	2	-	1 (2)	2 (4)	-
Highest*	0	19 (36)	-	18 (34)	30 (57)
	1	7 (13)	4 (7)	11 (21)	11 (21)
	2	20 (38)	14 (27)	18 (34)	7 (13)
	3	7 (13)	35 (66)	6 (11)	5 (9)

Abbreviations: *Pre-Tx* pre-treatment morbidity; * The highest morbidity/toxicity in a patient was counted as a single event; † During therapy and until 3 months after completion; ‡ maximal late toxicity > 3 months after completion of therapy; § Incidence of late toxicity at last follow-up visit. Percentages were rounded to reach 100% for every symptom.

The highest grade of acute toxicity was: grade 2 in 14 patients (27%); grade 3 in 35 patients (66%). No toxicity related interruptions of RT occurred. The highest grade of late toxicity was: grade 2 in 18 patients (34%); grade 3 in 6 patients (11%). At the last follow-up visit the highest grade of late toxicity was: grade 2 in 7 patients (13%); grade 3 in 5 patients (9%).

## Discussion

Since 2007 the outcome and patterns of failure of postoperative IMRT for OCC have been described by others [7-13].

Our patient selection equals those of the mentioned publications in regard to median age, TNM and AJCC stage, median follow-up and time interval between surgery and RT. Our CTV definitions and dose prescriptions are almost identical to those reported by Sher et al. [13], the relevant difference being that we prescribed 54 Gy to dissected and involved lymph node levels in the absence of ECE instead of 60 Gy. The CTV definitions and dose prescriptions of the remaining publications vary considerably in detail, especially when describing high-risk CTV. The FTB is uniformly assigned doses around 70 Gy in case of gross residual disease and 66 Gy in R1 except by Chen et al. [10] who applied a median dose of 61.1 Gy. The prescription range for dissected lymph node levels containing metastatic nodes is 60–66 Gy in the presence of ECE and 54–60 Gy without ECE, elective (low neck and contralateral) lymph node levels are prescribed 46.8 to 56 Gy.

The 2- and 3-year LRRFS, DMFS and OS estimates observed in our study compare well with previously published reports (Table 5). N-classification was the only significant prognostic factor for both LRRFS and OS in multivariate analysis, consistent with the findings of Chen et al. [10]. Others reported ECE or positive margins and total treatment time as significant [8,11].

**Table 5:** Treatment details and results of retrospective reports with postoperative IMRT in oral cavity cancer

**Report (reference)**	**n**	**Median age (years)**	**Stage (%)**	**Median Time from surgery to RT (weeks)**	**Systemic therapy (%)**	**Median follow-up (mts)**	**LRFS (%)**	**LRRFS (%)**	**OS (%)**
Studer (7)	28	61*	III (14)	nr	86	19	92#	nr	83#
			IV (54)						
Chen (10)	22	50	III (27)	nr	9	44	nr	64**	67Â¶
			IV (73)						
Gomez (9)	35	nr	III (26)	7	29	28	89**	77	74
			IV (54)						
Daly (11)	30	61	III (23)	6	60	38	67	53	60
			IV (53)						
Sher (13)	30	nr	nr	7	nr	nr	nr	91#	85#
Present analysis	53	60	III (13)	7	55	28	83	79	73
			IV (57)						

Abbreviations: *LRFS* local recurrence-free survival, *LRRFS* locoregional recurrence-free survival, *mts* months, *n* eligible patients, *nr* not reported separately, *OS* overall survival, *RT* radiotherapy,*mean age; #2 year actuarial estimates; three year actuarial estimates; **Crude rate.

Our patterns of failure analysis revealed an unusual multifocal recurrence pattern in 8 out of 12 patients involving areas of tumor resection, neck dissection and flap reconstruction making a precise allocation into categories of “local” or “regional” recurrence quite impossible. In relation to CTV54 and CTV60-66 or CTV72 coverage the recurrences showed an unexpected variety (Table 3) with “marginal” and “out” volume recurrences in relation to both CTVs. Four patients had multifocal recurrences completely covered by CTV54; therefore they could have been reported as “in” volume recurrences, but because less than 95% of all recurrent tumor masses were covered by CTV66 we additionally report them as “marginal” to CTV66. One of these patients for example recurred in the FTB and in the ipsilateral dissected and contralateral not dissected neck with 6 volumes of tumor all within CTV54, whereas 2 recurrences in the contralateral Level I were not covered by CTV60-66. Thus, in 10 out of 12 patients the locoregional recurrence occurred marginally or outside of the high-risk target volumes. A similar pattern of failure is reported by Yao et al. [8] with 4 out of 9 locoregional failures being multifocal and 3 of these 4 failing in both CTV60 and CTV64-66, though neither aspect is discussed in the paper. Daly et al. [11] give a detailed description of 13 locoregional treatment failures after postoperative RT, with only 3 failures “entirely within high-risk PTV60 or PTV66” and 2 multifocal failures not assessed in respect to PTV coverage at all, meaning the remaining 8 failures were at least “marginal” or “out” of the high-risk volume. The remaining publications however [7,9,10,13] do not quantify dose coverage of their failures and do not describe multifocal recurrences. We therefore propose to describe recurrences as unifocal or multifocal as this is an important predictor for surgical salvage [19].

Eight out of our 12 recurrences had extensive surgery requiring flap reconstruction, and 5 of these failed multifocally involving the flap reconstruction. All of these 5 patients had ECE, and there might have been tumor cell spillage during surgery [15]. Finding no proposals for target volume delineation regarding flap reconstructions in an extensive literature search we have now started to fully include flap reconstructions in the CTV54 in case of synchronous ECE.

Another one of our patients with extensive perineural invasion failed “out” of treatment volume having recurred in the ipsilateral infratemporal fossa with tumor cells spreading retrograde along the inferior alveolar nerve, a type of recurrence already described in detail [8]*.*

Concerning side effects, the acute toxicity grading was 2 in 27% of patients and 3 for 66%. The late toxicity grading was 2 in 34% of patients and 3 in 11%. The use of different methods for assessing toxicities and the variety of symptoms assessed makes it difficult to compare results across trials. However, our data do compare favorably with the toxicity rates outlined by Sher et al. assessed by CTCAE version 4.0. [13]. It is important to note that we did not observe acute toxicity related treatment interruptions. When late toxicity was assessed at the last follow-up visit, the rates were below the pre-RT morbidity symptoms. This could be partly due to increased pain medication, use of gastrostomy tubes and other medical interventions as well as full recovery from surgery. Our study is limited due to its retrospective nature. Despite this limitation it appears that LRRFS after postoperative IMRT is promising with acceptable acute and late toxicity rates.

## Conclusion

Postoperative IMRT of locally advanced or high-risk OCC was associated with satisfying LRRFS. Acute and late toxicity rates were acceptable. Our patterns of failure analysis emphasizes the need to improve high-risk CTV target volume definition, as the majority of our locoregional recurrences occurred marginal to or outside of the high-risk CTV. Having seen unusual multifocal recurrences in 5 of 8 patients with both ECE and flap reconstruction, we propose to fully include the volume of flap reconstruction in the elective CTV in this scenario. Further improvement of high-risk CTV definition might generally transfer into better LRRFS.

## Competing interest

The authors declare that there are no financial disclosures or conflict of interest that could be perceived as prejudicing the impartiality of the research reported.

## Authors’ contributions

Each author had participated sufficiently in the work to take public responsibility for appropriate portions of the content. AG, PG and DMA designed the study. SC performed the statistical analysis. AG, BB and PG collected the data and together with SC, AA, PM, WH and DMA interpreted the data. The manuscript was written by AG and PG, all other authors helped and finally approved the final manuscript.
